# Supplementary figures and images for: Spatial distribution of ticks and tick-borne pathogens in central Hokkaido, Japan and associated ecological factors revealed by intensive short-term survey in 2024
Source: PLoS One. 2026 May 14;21(5):e0349386. doi: 10.1371/journal.pone.0349386 (PMC13175462; doi:10.1371/journal.pone.0349386)

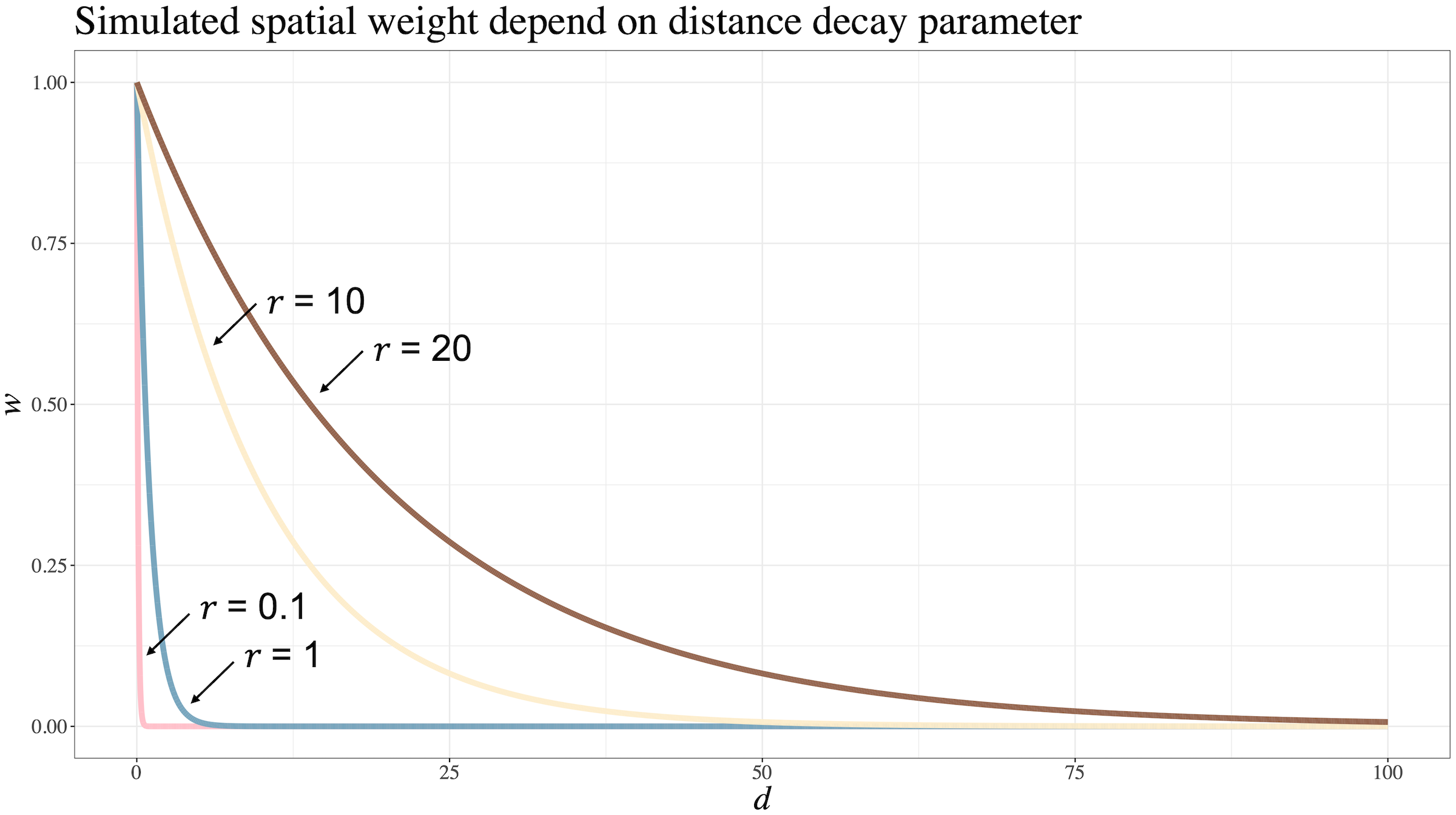

Supplement: S1 Fig — An element of spatial weights matrix (w in y axis), which is defined by the distance (km) between sites (d in x axis), was simulated by varying the parameter controlling the distance decay (r = 0.1, 1, 10, or 20). (TIFF) [file pone.0349386.s001.tiff]

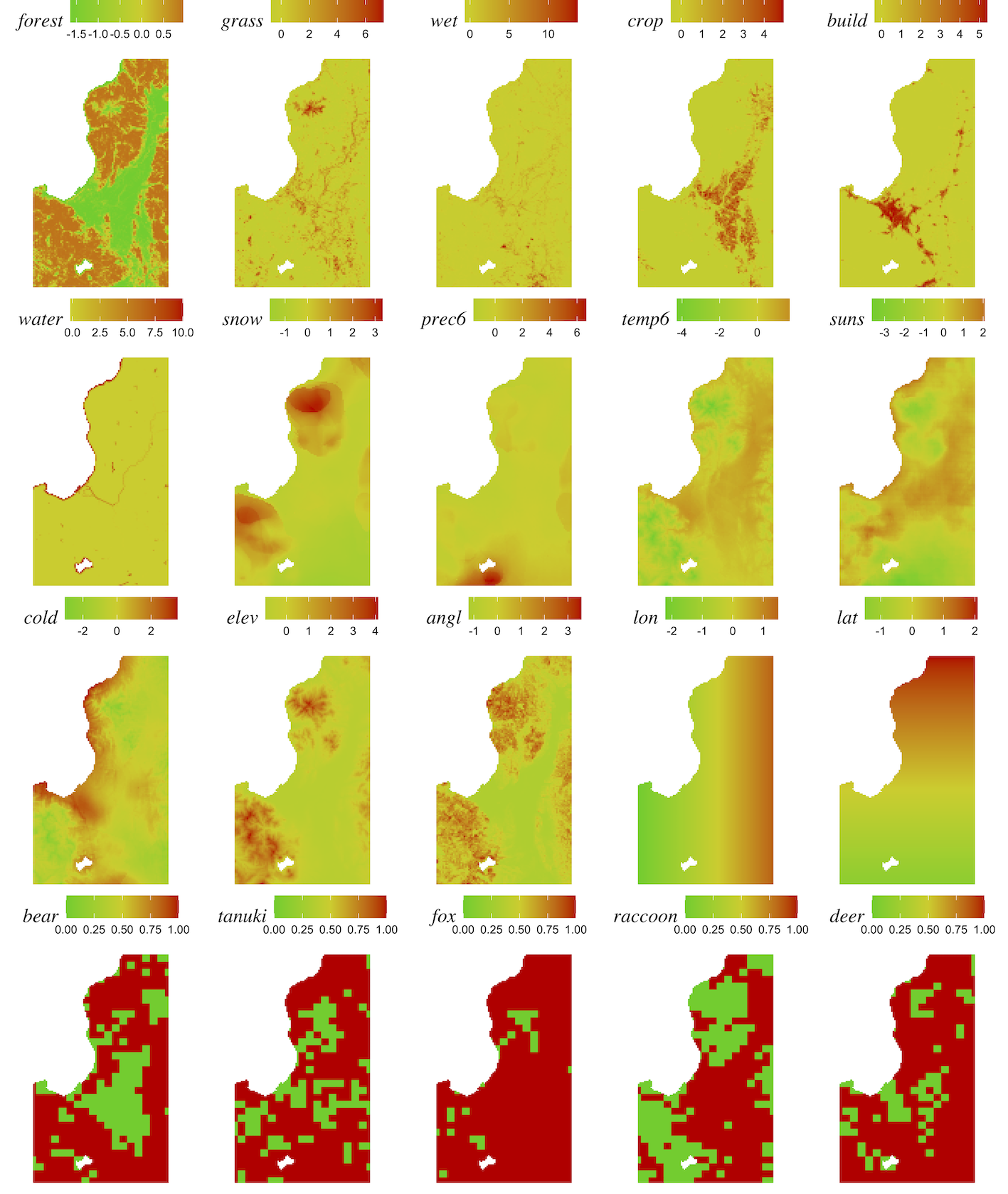

Supplement: S2 Fig — Distribution of each environmental variable was mapped across the study area. (TIFF) [file pone.0349386.s002.tiff]

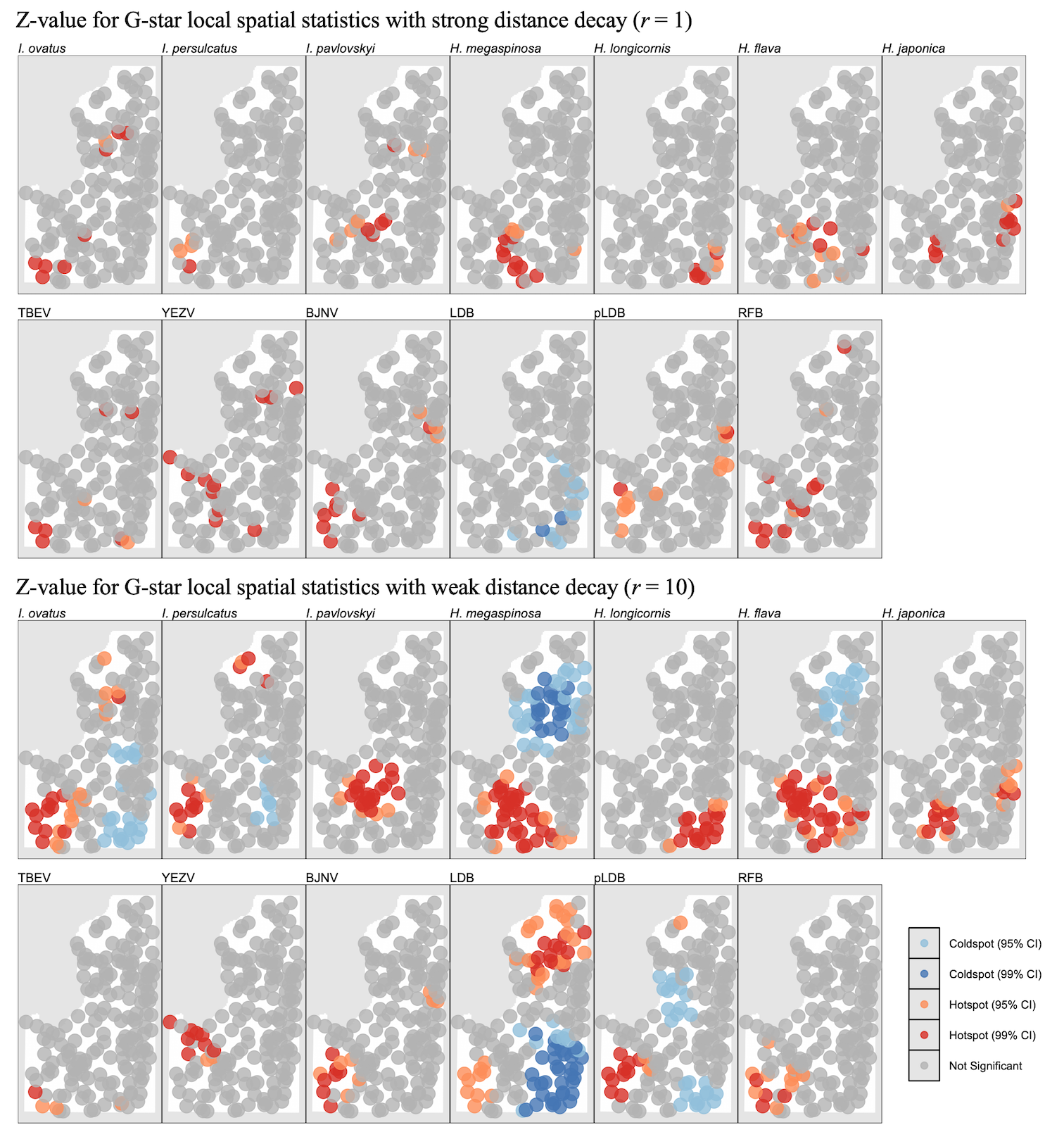

Supplement: S3 Fig — Z-values of each tick and pathogen for G-star local spatial statistics using different distance decay (r = 1 or r = 10) was indicated with different color: red, z-value > 2.58; orange, z-value > 1.96; light blue, z-value < −1.96; blue, z-value < −2.58; grey, −1.96 ≤ z-value ≤ 1.96. (TIFF) [file pone.0349386.s003.tiff]

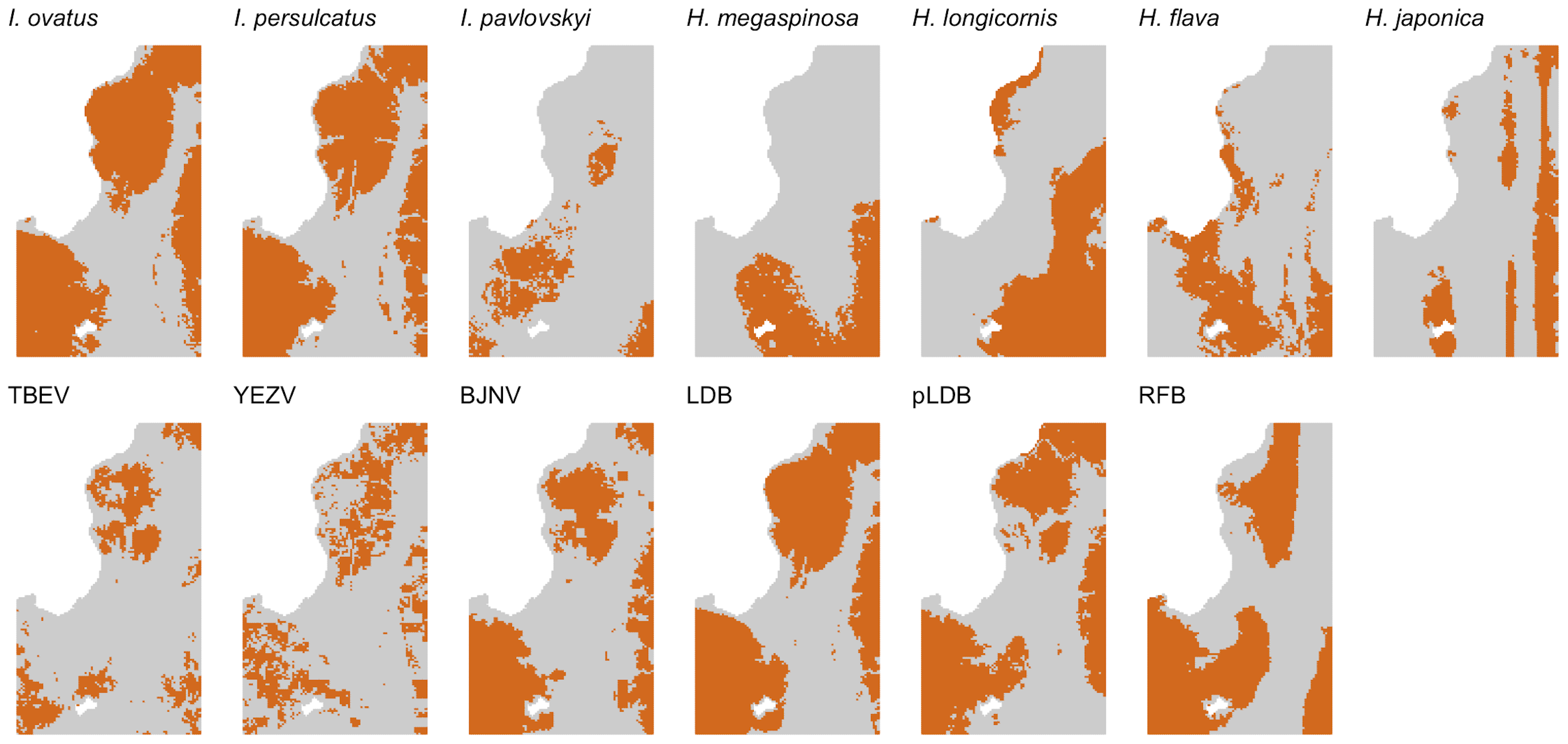

Supplement: S4 Fig — The presence probability was converted to presence/absence using the threshold which maximize TSS. Grids predicted to be present are indicated in orange, while grids predicted to be absent are indicated in grey. (TIFF) [file pone.0349386.s004.tiff]
