# Supplementary material for: Spatial distribution of ticks and tick-borne pathogens in central Hokkaido, Japan and associated ecological factors revealed by intensive short-term survey in 2024
Source: PLoS One. 2026 May 14;21(5):e0349386. doi: 10.1371/journal.pone.0349386 (PMC13175462; doi:10.1371/journal.pone.0349386)
Supplement: S3 Table — A present site of each tick species was defined as a site where one or more adult ticks were collected, and a present site of each pathogen species was defined as a site where one or more pools were tested positive. (DOCX) [file pone.0349386.s007.docx]

|  | Species | Number of present sites |  |
| --- | --- | --- | --- |
| Tick | *I. ovatus* | 139 |  |
|  | *I. persulcatus* | 97 |  |
|  | *I. pavlovskyi* | 10 |  |
|  | *H. megaspinosa* | 47 |  |
|  | *H. longicornis* | 22 |  |
|  | *H. flava* | 41 |  |
|  | *H. japonica* | 10 |  |
| Pathogen | TBEV | 7 |  |
|  | YEZV | 7 |  |
|  | BJNV | 9 |  |
|  | LDB | 117 |  |
|  | pLDB | 42 |  |
|  | RFB | 13 |  |
