# Supplementary material for: Spatial distribution of ticks and tick-borne pathogens in central Hokkaido, Japan and associated ecological factors revealed by intensive short-term survey in 2024
Source: PLoS One. 2026 May 14;21(5):e0349386. doi: 10.1371/journal.pone.0349386 (PMC13175462; doi:10.1371/journal.pone.0349386)
Supplement: S5 Table — AUC and TSS were calculated using leave-one-out cross-validation (LOOCV) or stratified 5-fold cross-validation. Results of Moran’s I test using r = 1 for the residuals of best model are shown in this table. (DOCX) [file pone.0349386.s009.docx]

|  |  | LOOCV | | Stratified 5-fold cross-validation | | | | Moran’s *I* test for residuals | |
| --- | --- | --- | --- | --- | --- | --- | --- | --- | --- |
|  | Species | AUC | TSS | AUC mean | AUC SD | TSS mean | TSS SD | Moran’s *I* | p-value |
| Tick | *I. ovatus* | 0.88 | 0.64 | 0.88 | 0.06 | 0.71 | 0.12 | -0.02 | 0.585 |
|  | *I. persulcatus* | 0.84 | 0.53 | 0.84 | 0.05 | 0.60 | 0.10 | 0.07 | 0.187 |
|  | *I. pavlovskyi* | 0.82 | 0.66 | 0.92 | 0.06 | 0.87 | 0.12 | -0.18 | 0.988 |
|  | *H. megaspinosa* | 0.90 | 0.71 | 0.91 | 0.05 | 0.78 | 0.08 | -0.01 | 0.496 |
|  | *H. longicornis* | 0.86 | 0.63 | 0.80 | 0.14 | 0.61 | 0.23 | -0.05 | 0.700 |
|  | *H. flava* | 0.71 | 0.41 | 0.57 | 0.13 | 0.26 | 0.23 | -0.02 | 0.586 |
|  | *H. japonica* | 0.80 | 0.58 | 0.79 | 0.22 | 0.72 | 0.28 | -0.02 | 0.558 |
| Pathogen | TBEV | 0.75 | 0.53 | 0.80 | 0.19 | 0.75 | 0.24 | 0.10 | 0.133 |
|  | YEZV | 0.72 | 0.52 | 0.89 | 0.08 | 0.85 | 0.11 | -0.02 | 0.578 |
|  | BJNV | 0.87 | 0.69 | 0.91 | 0.04 | 0.86 | 0.09 | -0.12 | 0.934 |
|  | LDB | 0.83 | 0.57 | 0.84 | 0.03 | 0.63 | 0.08 | 0.07 | 0.182 |
|  | pLDB | 0.86 | 0.59 | 0.78 | 0.10 | 0.57 | 0.12 | -0.02 | 0.548 |
|  | RFB | 0.76 | 0.61 | 0.71 | 0.14 | 0.61 | 0.16 | 0.11 | 0.068 |
