# Supplementary material for: Spatial distribution of ticks and tick-borne pathogens in central Hokkaido, Japan and associated ecological factors revealed by intensive short-term survey in 2024
Source: PLoS One. 2026 May 14;21(5):e0349386. doi: 10.1371/journal.pone.0349386 (PMC13175462; doi:10.1371/journal.pone.0349386)
Supplement: S1 Text — (DOCX) [file pone.0349386.s012.docx]

**Supplementary Text S1. Additional confirmation of RT-qPCR/qPCR positive samples.**

To examine the specificity of our screening methods, we performed additional confirmation of RT-qPCR/qPCR positive samples as detailed below.

For BJNV, all eleven RT-qPCR-positive samples were successfully confirmed by sequencing a 1,000-bp of partial RNA-dependent RNA polymerase gene in the L segment. The sequences showed >98% identity to the closest available BJNV sequences in GenBank (Table below). The PCR assay followed the protocol by Kishimoto et al. (2024). This result demonstrates the high specificity of our RT-qPCR assay targeting BJNV.

| Sample ID* | Species | Stage | Sex | The closest sequence (% Identity, Accession No.) |
| --- | --- | --- | --- | --- |
| 136 | *Ixodes* spp. | Nymph | - | Beiji nairovirus NE-YC4 (99.3%, ON408102) |
| 154 | *I. persulcatus* | Adult | Female | Beiji nairovirus SBC-4130 (99.9%, LC795509) |
| 155 | *I. persulcatus* | Adult | Male | Beiji nairovirus MNM-3915 (98.3%, LC795507) |
| 213 | *I. ovatus* | Adult | Female | Beiji nairovirus RUS-4138 (98.6%, LC795511) |
| 340 | *I. persulcatus* | Adult | Male | Beiji nairovirus RUS-4138 (99.6%, LC795511) |
| 579 | *I. persulcatus* | Adult | Female | Beiji nairovirus BS-321 (99.8%, LC815280) |
| 580 | *I. persulcatus* | Adult | Male | Beiji nairovirus BS-321 (99.8%, LC815280) |
| 604 | *I. persulcatus* | Adult | Female | Beiji nairovirus UTR-4157 (99.5%, LC795513) |
| 661 | *I. persulcatus* | Adult | Male | Beiji nairovirus RUS-4138 (99.3%, LC795511) |
| 789 | *I. persulcatus* | Adult | Female | Beiji nairovirus BS-1288 (99.3%, LC815291) |
| 905 | *I. persulcatus* | Adult | Female | Beiji nairovirus MNM-3915 (99.3%, LC795507) |

*Sample ID is the same with that in Supplementary Table S2

For TBEV, we attempted to sequence an approximately 250-bp fragment of the NS5 gene using the seven RT-qPCR-positive samples. Primers were 5′–TACAACATGATGGGAAAGAGAGAGAA-3′ and 5′–GTGTCCCAGCCGGCGGTGTCATCAGC–3′. One sample (Sample ID 657) was successfully sequenced and confirmed as TBEV (closest GenBank match: isolate A53/2023/China/Genhe, Accession No. PV183920). For the remaining six samples, amplicons could not be generated for sequencing, likely due to low viral loads and high sensitivity of the RT-qPCR assay (Achazi et al., 2011).

For YEZV, we tried to validate the eight N-gene-positive samples using a secondary RT-qPCR assay which targeting the RNA-dependent RNA polymerase gene using primer 1: 5′– CTCAACCTGCTTCCAACCTATC-3′, primer 2: 5′–GGTGTAAAGCCCAACATCCT–3′, and probe: 5′–/ 5Cy5/CCAAGGAAG/TAO/CACACAGATGGGTACA/3IAbRQSp/–3′. Two samples tested positive for this secondary RT-qPCR.

For the multiplex qPCR assay for LDB and RFB, we relied on the extensive validation performed by Takano et al. (2014), which demonstrated high sensitivity and specificity. Consequently, we considered our qPCR results for these pathogens reliable without additional sequencing.

In summary, while we cannot completely rule out the possibility of false positives among the unconfirmed high-Ct-value samples (e.g., for TBEV), treating these samples as negative would risk excluding true positives, thereby underestimating the pathogen's prevalence and reducing the statistical power of our prediction models. We believe that maximizing sensitivity is a justifiable approach for the aims of the present study.
