# Supplementary material for: Spatial distribution of ticks and tick-borne pathogens in central Hokkaido, Japan and associated ecological factors revealed by intensive short-term survey in 2024
Source: PLoS One. 2026 May 14;21(5):e0349386. doi: 10.1371/journal.pone.0349386 (PMC13175462; doi:10.1371/journal.pone.0349386)
Supplement: Attachment — Submitted filename: Response to Reviewers.docx [file pone.0349386.s013.docx]

**Point-by-point responses to reviewer comments**

We really thank the editor and three reviewers for thoughtful and productive suggestions that have helped in improving the manuscript. We also appreciate the production editors for supporting the review process. All changes to the manuscript are marked by “Track Changes” option in 'Revised Manuscript with Track Changes'. Please find our response to your comments below, where the line numbers indicate the line numbers in the clean copy without track changes ('Manuscript').

**Response to the requirements of Journal office:**

**CC BY 4.0 license for copyrighted images**

We have contacted the original copyright holder, ESRI, for use of the basemaps in Figures 1, 2, and 4 under the CC BY 4.0 license. Since it may take a month to receive the official letter, we’d like to submit the revised manuscript as it is. We will replace the basemaps with the appropriate license, if ESRI rejects our request.

**Data in the Supporting Information file <Tabel S1>**

Our data do not include any identifying data nor human data across the manuscript and supporting information. Supplementary Table S1 only includes the number of collected ticks and pathogen-positive ticks at each field site. Besides, location information, including coordinates of each site, site ID, and address, are not included. We would like to publish this table to response to the comment 7 by Reviewer #1.

**Restrictions for sharing data**

Due to the ethical policy for sharing the exact locations of sampling, there is a restriction to data sharing for this study. All the other data except for geographical coordinates are fully available in the manuscript and supporting information. This situation is described in ‘Data availability’ statement in the manuscript and the online submission form.

**Separate files for tables in the manuscript**

All the tables are already included in the main manuscript (i.e., ‘Manuscript’ and ‘Revised Manuscript with Track Changes’). However, one of the reviewers required us to show larger table for review process (comment 21 and 23 by reviewer #2), and we’re not be able to address this request without uploading separate files. Thus, we attached two tables, which are already included in the main manuscript, as ‘Other’ file.

**Response to the comment of Academic editor:**

*The reviewers have completed their evaluation of your manuscript. I encourage you to revise and resubmit your work, ensuring that all reviewer comments are thoroughly addressed. Please incorporate the feedback carefully and provide a detailed, point-by-point response that clearly outlines every change made in response to the reviewers’ suggestions.
In addition, kindly correct all typographical and grammatical errors, and ensure that the manuscript is prepared in full compliance with the journal’s formatting and submission guidelines.
We look forward to receiving your revised submission.*

**Response:** We appreciate the editor’s contribution to our manuscript. We have completed the revision of the manuscript and response to the reviewer’s comment as follows. In addition to the revisions incorporating feedback by the reviewers, we carefully checked the submission guidelines of your journal to revise our manuscript. Typographical and grammatical errors were also corrected.

**Response to the comments of Reviewer #1:**

1. *The introduction section is too general; introduce specific tick species, relevant pathogens, and their public health relevance earlier. Add a proper gap statement: prior Hokkaido work has patchy coverage, and this study contributes tick, pathogen data, and comparative ENMs.*

**Response:** We revised the third paragraph in introduction section to explain the detail situations of Hokkaido, including specific tick species and their relevant pathogens, and to clearly state a knowledge gap (lines 86-95). Besides, for responding to the other reviewers’ suggestions, we retained the first and second paragraph for broad readers of the journal.

2. *Strong on geography and effort standardization. However, using a single collector and a fixed 30-minute effort per site, during a short period of 43 days, could bias detectability, so acknowledge this as a limitation.*

**Response:** We agree with this comment and mentioned it as one of the limitations of the present study in the discussion section (lines 589-592).

3. *Following Moran’s I, conducting Hotspot analysis & Cluster outlier analysis will point out the regions of hotspots, and add confidence to it.*

**Response:** Thank you for your suggestion to strengthen our spatial analyses. Following the reviewer’s suggestion, we additionally calculated the local spatial statistic G-star to point out hotspots (lines 244-249). The result was described in result section (on lines 431-435) and z-sores were visualized in Supplementary Fig. S3. The caption for Fig. S3 is on lines 833-837. The pointed out hotspots generally aligned with the mapped raw data on Fig. 2.

4. *Provide AUC/TSS distributions (median, IQR) across species*

**Response:** The median and IQR of AUC and TSS across species were provided on lines 442-444.

5. *In discussion, don’t overstate “risk” as it creates conflicts with modeled suitability for human incidence. Please define outputs as the presence probability of infected ticks, because human behavior exposure, seasonality across the year, wasn’t measured, which limits how directly results translate to cases.*

**Response:** We followed this suggestion and revised the sentences lines 509-510, 513-516, and 522-525 to erase the word “risk.” In addition to the reviewer’s suggestion, we also referred to comment 24 by reviewer #2 and revised the first paragraph of discussion section (lines 510-516) to improve clarity.

6. *Close the discussion section with two or three concrete next steps and a single sentence “so what” message, so the Discussion ends with a clear take-home.*

**Response:** We appreciate the reviewer’s suggestion to improve our manuscript. We added one paragraph at the end of discussion section, where the future prospects were described (lines 600-606).

7. *Consider releasing data, full raster outputs, and code to enable reproducibility while protecting sites.*

**Response:** All the explanatory variables in ecological niche modeling were gained from open data, which readers can freely obtain from each recourse. The response variables in the ecological niche modeling and data for spatial analysis, except for their coordinates, were shown in Supplementary Table S1, which was mistakenly removed from the initial submission. In this study, our variable selection include an iterative manual process where we made decisions at each step based on a simultaneous assessment of multiple metrics (AUC, TSS, VIFs, and Moran’s *I*). Therefore, to ensure the greatest clarity, we have provided a detailed, step-by-step description of the criteria and the selection process in the method section (lines 304-339). We believe this detailed description is more informative and transparent than a script of the manual selection.

8. *Line 136-138: More information on pools, how many pools in total, and species or genus-wise?*

**Response:** Since we thought that only the pooling strategy should be mentioned in the materials and methods section, the actual information of pools has been described in the results section. The number of pools in total (1026 samples) was explained in the second paragraph of the result section (lines 377-378) and species/genus-level information was summarized in Table 2. The medians of pooled individual ticks per species/genus were added in Table 2 to follow the reviewer’s comment 9.

9. *L144–198: Pooling rules are clear; add median pool sizes per species/stage and max Ct acceptance rationale. How non-amplifying pools were handled.*

**Response:** Following the reviewer’s suggestion, we added the medians of pooled individual ticks per species/genus in Table 2.

The max Ct acceptance (< 45) was to maximize the sensitivity of our screening, aiming to capture as many potential positives as possible for our ecological niche modeling. Since we believe the genetic detections are the most sensitive methods, negative samples in each RT-qPCR/qPCR were treated as negative. Regarding the specificity of the screening methods, we performed additional examinations including sequencing of fragments. Please kindly refer to the detail results of our additional examinations provided in our response to the comment 29 by the reviewer #3. We described the rationale and validation results as a limitation of our study on lines 592-597.

10. *Line 221-223: Moran’s I being -1 should be interpreted as negative spatial autocorrelation with neighbors being very different from each other.*

**Response:** Thank you for reading the detail of our manuscript. According to the reviewer’s suggestion, the interpretation of Moran’s *I* value was revised as follows: “Moran’s *I* close to –1 means that neighboring sites are different from each other, while Moran’s *I* close to 1 means that the species distribute in cluster.” on lines 240-242.

11. *L226–291: GLM with LOOCV is transparent, but presence/absence derived from short-term sampling risks false absences, and LOOCV can be spatially optimistic. At least add a sensitivity analysis. Explain the VIF threshold and any variable interactions.*

**Response:** Following the reviewer’s suggestion to add a sensitivity analysis, we additionally performed a stratified 5-fold cross-validation to evaluate the stability of the model's predictive performance. The methods were described on lines 339-344 and the results were explained on lines 444-449 and supplementary Table S4. The means of AUC and TSS calculated by stratified 5-fold cross-validation were above acceptable thresholds for predictions (AUC > 0.7 and TSS > 0.5) and their standard deviations were small, except for *H. flava*. We agree with the reviewer’s concern associated with our sampling and modeling methods, and the limitations were described in discussion section (lines 589-592 and 597-602).

Explanations on VIF and variable interactions were added on lines 326-329 and 308-310. In short, VIF threshold was set at 5 and no interaction terms of environmental variables were considered in order to avoid complexity in modeling.

12. *L226–256: There’s a scale mismatch (mammals 5×5 km, others 1×1 km). Discuss potential smoothing bias or add a sensitivity analysis without mammal layers.*

**Response:** For the mammal variables, the data from each 5×5 km grid was assigned uniformly to all 25 of the 1×1 km grids nested within it (i.e., a block assignment was performed instead of smoothing). We revised the sentence to explain the assignment method of mammal distribution on lines 294-295. We also acknowledge the reviewer’s concern that this approach assumes a uniform distribution of the mammal species within each 5×5 km grid. The limitations associated with the scale mismatch was added in the discussion section (on lines 542-547).

**Response to the comments of Reviewer #2:**

13. *The concept of a reservoir—both for ticks (lines 47–50) and for hosts (lines 54–58)—is never clearly mentioned, yet it is essential for understanding the circulation of potentially pathogenic microorganisms. This should be defined in the introduction.*

**Response:** We appreciate the reviewer’s advice to improve our manuscript. Following the reviewer’s suggestion, we revised the sentences to clearly use the word “reservoir” on lines 47-49 and 54-56.

14. *Lines 80–81: In Europe and the USA, nymphs are generally considered the highest risk. Why would adults be more at risk in Japan?*

**Response:** We understand the reviewer’s wonder at the regional difference. At least, local epidemiological data from Hokkaido show that the most tick bites are caused by adult ticks; Miyamoto et al. (1991) reported that all of 58 tick bites were caused by adults and Sasaki et al. (2021) reported that only one out of 153 tick bites was caused by nymphal tick. Based on this local situation that adult ticks are the main vector for humans in this region, our research was designed to focus on them. We revised the sentence on lines 86-89 in introduction section to more clearly emphasize this specific epidemiological situation in our study area.

15. *Line 95: Consider removing the adjectives “intensive” and “comprehensive” and let the reader form their own judgment.*

**Response:** According to the reviewer’s suggestion, we revised the sentence to erase the abstract words and to make the concrete explanation in introduction section (lines 98-100).

16. *Tick collection method: Typically, tick density is measured per unit area (e.g., per 100 m²) rather than per unit time. Why did the authors choose a 30-minute duration, which seems short? What approximate distance was covered during this time?*

**Response:** Indeed, we don’t aim to determine tick density in the present study. Because of the topographical diversity in our study area, we were not able to set an area in which a person could freely move for flagging. Furthermore, we prioritized increasing the spatial coverage across the study area by sampling at a larger number of sites. We selected a time-based sampling since it is more robust and convenient to standardize the sampling effort in our study area. In response to your question, the approximate distance covered during the 30-minute period was 750 meters. We added the sentence on lines 141-143 to explain the reason why we selected a 30-minute duration for flagging. We also acknowledge the reviewer’s concern that a 30-minute flagging was short and might miss minor tick species at each site, and described this limitation in discussion section (lines 589-592).

17. *Tick analysis method: It appears that pools were used to test both nymphs and adults, especially for virus detection in ticks. For bacteria, a pool of 100 nymphs is extremely large. While this is explained later in the text, the “Materials and Methods” section should specify that only presence/absence was ultimately measured.*

**Response:** We added a sentence on lines 217-219 in materials and methods section to emphasize the data of pathogen screening was used as presence/absence data in the subsequent spatial analyses. For readability, the sub-sections “Nucleic acid extraction and pathogen detection” and “Definition of presence/absence of each tick/pathogen at a site” were retained separately.

18. *Lines 193–198: It is not clear how the ticks were genotyped for Borrelia. How were the Borrelia species identified—by genotyping targeting a specific gene, or by sequencing?*

**Response:** We agree that detailed genotyping of *Borrelia* is important for molecular epidemiological studies as performed in multiple studies from our study area (Nakao et al., 1994; Masuzawa, 2004; IDSC, 2011; Takano et al, 2014; Okado et al., 2021), which were also cited in the manuscript. The aim of our study was not to reproduce the species specificity of individual genotypes, but rather to understand the spatial distribution of the major species groups. To achieve this, we used the multiplex qPCR assay from Takano et al. (2014), which is well-validated for specifically distinguishing *Borrelia burgdorferi* s.l. (LDB) from *B. miyamotoi* (RFB). Since the previous studies cited above have indicated that LDB specifically carried by *I. persulcatu*s are causing Lyme disease in our study area, we treated LDB detected in *I. persulcatus* (referred as pLDB) as a separate variable during the statistical analyses in addition to the total LDB. We revised the sentences on lines 205-213 to clarified this rationale.

19. *Lines 200–204: This section is unclear. Please rephrase for clarity.*

**Response:** We added a sentence in this section for clearly explaining the definition to convert the data of tick collection and pathogen detection to presence/absence data for the subsequent statistical analyses (lines 217-219).

20. *Many abbreviations are used without being defined (e.g., pLDB on line 198, and throughout the Ecological Niche Modeling section: prec6, temp6, elev, angl, etc.). While these are explained in Table 1, they should also be defined in the text. I recommend moving Table 1 to the supplementary data.*

**Response:** Thank you for your comment. We spelled out all the abbreviations pointed out by the reviewer at the first appearance in the text (lines 211-213, 254-256, 261-265, and 287-291). For easier understanding of modeling method, we retained Table 1 in the main manuscript.

21. *Increase the resolution of Table 2 by enlarging the font size.*

**Response:** The font size of Table 2 was enlarged following the reviewer’s comment. We also attached an Excel file named “Table2&4forReview”, which includes Table 2 for efficient review process.

22. *Lines 378–380: Why were hosts not included in the analysis, given that they are essential for ticks, which are strictly hematophagous?*

**Response:** All the tick species appeared in the manuscript are known as hematophagous. We further discussed the reasons why mammal variables were rarely included in the best model (lines 542-547). Please also refer to the response to your comment 26, where we explain the reason in other words.

23. *Table 4: Increase the resolution of the table by enlarging the font size.*

**Response:** The font size of Table 4 was enlarged following the reviewer’s comment. Please also find an attached Excel file named “Table2&4forReview”, which contains 4 for efficient review process.

24. *Lines 431–439: It is unclear what is actually being discussed here. Please revise. Previous studies have used presence/absence data, as in the present study. Other studies on human cases and serology in wildlife are also important and should be addressed.*

**Response:** We agree with the reviewer’s comment that occurrences of diseases and serological data from wildlife are also important. In this section, we want to claim that short-term intensive tick collections can be used for revealing the local distributions of ticks infected with endemic pathogens. This information, in turn, should help to understand infection risks from a different perspective than disease and serological data. We revised the sentences on lines 510-516 to clarify that we can use the data of ticks infected with pathogens as well as other data for revealing the spatial distribution of infection risk.

25. *Lines 452–453: It would be interesting to develop this argument further. Why does snow cover impact tick distribution? Why would Ixodes be more affected by this factor compared to Haemaphysalis?*

**Response:** We added the discussion on the interaction between snow and tick (lines 528-535). In contrast to strong impacts by snow depth on ticks through winter survival or the abundance of preferential host, the distributions of pathogens seemed not to be affected by snow depth.

26. *Lines 460–461: I am truly surprised by this observation. Wildlife plays an essential role in the presence and maintenance of ticks in a given environment. Please clarify and rephrase.*

**Response:** We agree with the reviewer’s comment. Wild mammals shall play a fundamental role in the tick life cycle. We believe the small number of significant associations in our model is attributed to the limitations of the available mammal distribution data, rather than a lack of the true ecological interactions. The available data in our study area were coarse (5 × 5 km grid) presence/absence records. This means that a single "presence" value was assigned to a 25 km² area, failing to capture the fine-scale variations of mammal density, which should be more direct driver of tick life cycle. Consequently, the mammal variables likely lacked sufficient variance at our analysis in 1 × 1 km to statistically explain the tick/pathogen distribution. To properly investigate the interactions between mammal and tick/pathogen species, their density data at a finer spatial resolution would be required. We revised the sentences on lines 542-547 to explain this limitation of the present study.

27. *Lines 483–484: I understand that ticks infected with viruses may be clustered, but this is generally not the case for Lyme Borrelia and relapsing fever Borrelia. Please clarify.*

**Response:** We examined the spatial clustering in local to regional scale (i.e., approximately 1 km to 50 km in radius). In detail, we performed Moran’s *I* test using a spatial weights matrix (*w*) with a distance decay parameter (*r* = 0.1 to *r* = 20) as visualized in Supplementary Fig. S1.

Actually, Moran’s *I* for *Borrelia* spp. was significantly positive independent of the distance decay parameter (Supplementary Table S3). Thus, we may need to say that ticks infected with *Borrelia* spp. are clustered in our study area. Several studies from geographically distinct regions have also reported the clustering of *Borrelia*-infected ticks (Kjær et al., 2020, 10.1038/s41598-020-76334-5) or Lyme disease occurrence (e.g. Chen et al., 2005, 10.1093/jmedent/42.5.899; Zanzani et al., 2019, 10.1016/j.ttbdis.2019.07.001) in this spatial scale. We revised the sentence on lines 564-566 to clarify the spatial scale in our analysis for avoiding confusion.

**Response to the comments of Reviewer #3:**

28. *Sampling Design: The authors sampled adult and nymphal ticks at 171 sites from 13 May–26 June 2024 using a standardized 30-minute flagging protocol by a single collector. This consistent, high-effort sampling across a broad area is a strength, yielding 4,608 adult and 5,300 nymph ticks from 159 sites (Table 2). The sample size is impressive and likely captures much of the regional tick diversity in spring. However, the short study period covers only late spring–early summer; tick phenology in Hokkaido may extend into autumn, and different species or life stages (e.g. larvae) might be active later. The authors should acknowledge this seasonal limitation. Site selection is not described in detail: how were the 171 locations chosen (e.g. random, stratified by habitat)? Clarification of site selection would strengthen confidence that sampling was representative and not biased toward easily accessible areas.*

**Response:** Thank you for your understanding of our study design. The limitation associated with our sampling design was also pointed out by reviewer #1 (comment 2 and 11) and reviewer #2 (comment 16). We described this limitation in discussion section (lines 589-592).

Our site selection was random, while we intentionally avoid several regions where tick collection is prohibited by law. To confirm randomness of our investigated sites, we additionally performed an average nearest neighbor analysis in Arc GIS pro. As shown in the attached output file below, our 171 sites were randomly located. We added the sentence to explain our site selection on lines 134-136 and the method and result of average nearest neighbor analysis on lines 136-138 and 367-368.

29. *Pathogen Detection: Molecular screening used published RT-qPCR/qPCR assays for each pathogen. The authors provide primer/probe details and thermal cycling conditions (Methods). Using published, specific assays (e.g. Achazi et al. 2011 for TBEV, Nishimura et al. 2022 for Yezo virus) is sound. One concern is the high Ct cutoff (<45) for positives; this may risk false positives at the very end of amplification. It would help if the authors commented on assay sensitivity/specificity (for example, whether positive controls and negative controls were included) or if any PCR products were confirmed by sequencing to rule out non-specific amplification. Including standard validation (e.g. replicates, sequencing representative amplicons) would strengthen confidence in the results.*

**Response:** We acknowledge the potential risk of false positives with the Ct cutoff. Our choice of Ct < 45 was a deliberate decision to maximize the sensitivity of our screening, aiming to capture as many potential positives as possible for our ecological niche modeling, while acknowledging the trade-off with specificity. To validate our screening and address the reviewer’s concern, we performed additional confirmatory examinations and revised the manuscript as follows.

For BJNV, all eleven RT-qPCR-positive samples were successfully confirmed by sequencing a 1000 bp of partial RdRp gene in the L segment, showing > 98% identity to the closest BJNV sequences available in the GenBank. The PCR assay was referred to Kishimoto et al. (2024). This result shows high specificity of our RT-qPCR assay for this pathogen.

For TBEV, we attempted to sequence an approximately 250 bp fragment of the NS5 gene using the seven RT-qPCR-positive samples. Primers were 5′–TACAACATGATGGGAAAGAGAGAGAA-3′ and 5′–GTGTCCCAGCCGGCGGTGTCATCAGC–3′. One sample was successfully sequenced and confirmed as TBEV. For the remaining six samples, amplicons could not be generated for sequencing, likely due to a low viral load and high sensitivity of the RT-qPCR assay (Achazi et al., 2011).

For YEZV, we tried to validate the eight N-gene-positive samples by another RT-qPCR assay which targets the RdRp gene using primer 1: 5′– CTCAACCTGCTTCCAACCTATC-3′, primer 2: 5′–GGTGTAAAGCCCAACATCCT–3′, and probe: 5′–/ 5Cy5/CCAAGGAAG/TAO/CACACAGATGGGTACA/3IAbRQSp/–3′. Two samples tested positive for the RT-qPCR. One of these was subsequently confirmed through whole-genome sanger sequencing using 45 primers.

For the multiplex qPCR assay for LDB and RFB, we relied on the extensive validation performed by Takano et al. (2014), which demonstrated high sensitivity and specificity. Furthermore, we confirmed with the authors that false positives have not been an issue when using this assay on tick samples (Dr. A. Takano, personal communication). Therefore, we considered our qPCR results for these pathogens reliable without additional sequencing.

In summary, while we cannot completely rule out the possibility of false positives among the unconfirmed high-Ct-value samples (e.g., for TBEV), treating these samples as negative would risk discarding true positives, thereby underestimating the pathogen's prevalence and weakening the statistical power of our prediction models. We believe that maximizing sensitivity is an effective approach for the aims of the present study. We described this rationale and these validation results as a limitation of our study in the manuscript (lines 592-597) and retained the original Ct cutoff value.

30. *Statistical Modeling (Ecological Niche Models): The methods section lacks detail on the ENM procedure. It is not specified how absences or background data were chosen (e.g. were non-detection sites treated as absences?), nor which software or R packages were used. The variable selection process is unclear – did they start with all 18 covariates and use stepwise selection? Were collinearity or variance inflation assessed? The brief mention of applying “spatial smoothing” for some models is not explained. For reproducibility and rigor, the authors should provide more information: the modeling algorithm (e.g. GLM with binomial link), criteria for choosing best-fit models, and any preprocessing (e.g. standardizing variables, assessing correlations). As noted in the literature, SDMs for ticks require careful standardization and reporting. The manuscript would benefit from clarifying these steps. If possible, sharing the model code or parameters (e.g. in a supplementary file or repository) would help transparency.*

**Response:** The explanation on presence/absence data was added on lines 217-219. Following the reviewer’s suggestion, software or R package was cited immediately after the explanation of each analysis (lines 295-296, 303-304, 325-326, 330-333, and 349). Our variable selection include an iterative, manual process where we made decisions at each step based on a simultaneous assessment of multiple metrics (AUC, TSS, VIFs, and Moran’s *I*). Therefore, to ensure the greatest clarity, we have provided a detailed, step-by-step description of the criteria and selection process in the Methods section (lines 304-339). We believe this detailed description is more informative and transparent than a script of the manual selection would be.

31. *A major concern is the small number of positive samples for some pathogens. For example, only 7 pools were positive for TBEV and 8 for Yezo virus. Fitting a reliable habitat model on such few presences is challenging and may lead to overfitting. The authors should discuss this limitation. In particular, note that with many zeros (true negatives) and few positives, standard logistic regression may produce unstable coefficients. Did the authors aggregate pathogens into genus-level (e.g. “all Borrelia”) or model each pathogen separately? It appears each pathogen was modeled individually (Table 4 shows separate D/I values), which could be under-powered for rarer pathogens. More discussion of model confidence is warranted. Despite this, the authors do report model performance metrics, which suggests at least some predictive signal.*

**Response:** Modeling was separately performed for each pathogen (lines 304-306). We agree with the reviewer’s concern about model stability and additionally performed a stratified 5-fold cross-validation to evaluate the variance of the models' predictive performance. The means of AUC and TSS were above acceptable thresholds for predictions (AUC > 0.7 and TSS > 0.5) and their standard deviations were small, except for *H. flava*. The methods were described on lines 339-344 and the results were explained on lines 444-448 and supplementary Table S4. The limitation pointed out by the reviewer (i.e. the small number of presence) was described in discussion section (lines 597-602).

32. *Environmental Covariates: The inclusion of habitat (forest, grassland), topography (elevation), climate (temperature, snow depth) and host presence is comprehensive. The finding that annual maximum snow depth strongly influenced tick distributions (positively for Ixodes ticks, negatively for Haemaphysalis) is plausible given Hokkaido’s climate. However, snow depth did not enter pathogen models. The manuscript suggests this may indicate complex “interactions” between snow and pathogen cycles. This interpretation is reasonable but a bit speculative; it might reflect that pathogens depend on host factors not captured by snow alone. The negligible effect of wild mammal distributions in models is likely due to the coarse (5×5 km presence/absence) host data. The authors correctly note that finer-scale host data are needed to assess these effects. Overall, the choice and handling of covariates seems sound, but more detail on how each was processed (e.g. how snow depth was measured or averaged) should be added.*

**Response:** According to the reviewer’s comment, we expanded the discussion about interactions with snow depth. We cited several studies to explain the possible mechanisms of positive/negative effects on ticks (lines 528-535). Snow depth seems to increase overwinter survival of *Ixodes* spp. ticks, while it can decrease the abundance of sika deer, which is a crucial host for *Haemaphysalis* spp. ticks. We also revised the sentences on lines 542-547 to clarify the reasons of the small effects of mammal distribution variables in models.

The explanations how environmental data were measured were added on lines 260-261 and 268-271. All the environmental variables were acquired from open data source. As for climatic factors including snow depth, the values were the mean based on 30 years of meteorological observations by Japan Meteorological Agency.

33. *Statistical Analysis: Beyond ENMs, the authors calculated Schoener’s D and Warren’s I to quantify niche overlap between each pathogen and each tick species (Table 4). This is appropriate for comparing spatial distributions. They also computed Moran’s I for clustering. These analyses are straightforward and correctly interpreted (e.g. low overlap D between most viruses and ticks implies distinct distributions). Their results that I. persulcatus and I. ovatus have the highest overlap with all pathogens (D,I up to ~0.95 for LDB) are consistent with prevalence data (see below). Statistical methods are adequately applied, but the manuscript should ensure that all reported p-values and thresholds are explained (e.g. what is a “significant” Moran’s I).*

**Response:** We appreciate your positive assessment of our statistical approach. As suggested, the threshold of p-value (p-value < 0.05) was explicitly stated on lines 242-243 and 317-318.

34. *The authors’ interpretation of results is generally sound and well-supported by the data. Overall, the conclusions follow logically from the results. One area for improvement is to explicitly acknowledge the limitations of pathogen sample sizes when interpreting model outputs. For instance, predictions for TBEV and Yezo virus (each <10 positives) should be treated with caution. But the authors generally provide a balanced discussion, citing relevant literature (e.g. stable TBEV foci vs emergent YEZV foci) to contextualize their findings.*

**Response:** Following the reviewer’s suggestion, we added one more paragraph to describe the limitations of our study including the small positive sample sizes (lines 597-602).
