# Supplementary material for: Spatial distribution of ticks and tick-borne pathogens in central Hokkaido, Japan and associated ecological factors revealed by intensive short-term survey in 2024
Source: PLoS One. 2026 May 14;21(5):e0349386. doi: 10.1371/journal.pone.0349386 (PMC13175462; doi:10.1371/journal.pone.0349386)
Supplement: Attachment — Submitted filename: Response_to_Reviewers_auresp_2.docx [file pone.0349386.s015.docx]

**Point-by-point responses to reviewer comments**

We really thank the consideration by editor and reviewers for our manuscript. All changes to the manuscript are marked by “Track Changes” option in 'Revised Manuscript with Track Changes'. Please find our response to your comments below, where the line numbers indicate the line numbers in the clean copy without track changes ('Manuscript').

**Response to the comment of Academic editor:**

1. *In particular, Reviewer #3 has included several comments suggestions in the annotated manuscript. Please reply to those concerns.*

**Response:** Thank you for providing the opportunity to revise our manuscript. In accordance with Reviewer #3’s comments and the suggestions in the annotated manuscript, we have thoroughly revised the manuscript. We provide point-by-point justifications in this file for the comments for which we consider a detailed explanation is necessary. All other minor changes (e.g. grammatical corrections) requested in the annotated file have also been incorporated and replied to all of the reviewer #3’s comments in the annotated file (“Manuscript_replied.pdf”).

**Response to the comments of Reviewer #3:**

2. *The manuscript presents valuable data and a well‑structured ecological modeling framework; however, several important methodological, statistical, and reporting issues remain and require substantial revision before the study can meet PLOS ONE standards. Detailed, line‑by‑line and section‑by‑section comments have been provided in the attached document, including notes on sampling design limitations, PCR confirmation procedures, spatial cross‑validation, model interpretation, data availability, and English‑language clarity. Please address all points in the attached review document in your next revision..*

**Response:** Thank you for your tremendous contribution to our manuscript. We greatly appreciate the time and effort you invested in providing detailed, line-by-line suggestions in the annotated document. We have carefully revised the manuscript in accordance with the comments and replied to each of your comments individually within the attached file (“Manuscript_replied.pdf”). For the major comments requiring clarification, our detailed justifications are also provided below.

3. *Add one sentence of scope/results:*

**Response:** We agree with your suggestion to bridge the methodology and the results here. To achieve this, we have added a sentence describing our results of tick collection and pathogen detection and modified the above/following sentences on lines 29-35 to fit in the context. The revised sentences summarize the scope of our collection, initial screening results, and modeling outcome.

4. *Missing controls: time‑of‑day, weather/ground moisture, vegetation height/density—all affect catchability. Please report these covariates or acknowledge as sampling bias.*

**Response:** According to your suggestion, we have now specified the sampling conditions in the Materials and Methods section (on lines 141-142 and 144-147). Specifically, we clarify that we collected ticks during daylight hours on days with no precipitation across various habitat types. In addition, the sentence in Discussion to mention our limitation of sampling was also revised (lines 613-616).

5. *One 30‑min pass per site risks false absences. Consider an occupancy/detection sensitivity paragraph in the Discussion.*

**Response:** We revised the last paragraph in the Discussion to incorporate this limitation together with the above your comment #4 (line 613-616). Since this issue is closely associated with pathogen detection method and our modeling approach, we integrated this discussion into the existing paragraph to provide a more cohesive explanation of the study's limitations, rather than creating a separate section.

6. *“Samples with Ct<45 and exponential curves were considered positive”; partial sequencing only; no technical replicate reporting; no stated negative‑control outcomes. Action: Report (i) replicate policy, (ii) negative‑control results, (iii) confirmation rate by sequencing per pathogen, and (iv) GenBank accession IDs where available. This is essential given several positives at Ct > 39.*

**Response:** Thank you for pointing out these essential details. To address your concerns, we have revised the sentences in the Materials and Methods (lines 204–211) to explicitly state that all assays were performed in technical replicate with positive and no-template negative controls, and no amplification was observed in any negative controls. Furthermore, we have provided Supplementary Text S1, where further confirmation by alternative PCR assay, sequence data, and detailed justifications are provided. We have now cited this Supplemental information on line 395-397 in the Result section.

7. *“Tick present if ≥1 adult collected; pathogen present if ≥1 positive pool.” This excludes nymphs from tick presence despite being numerous and ecologically relevant. Please justify (morphological limits are fine) and discuss consequences for ENM.*

**Response:** We used this definition to ensure the highest accuracy of morphological identification. The sentence was added on lines 231-232 to explain this point. In addition, the sentence to explain the definition (on lines 229-231) were revised for readability.

8. *Provide the rationale for the reported r values in Table 3 (you show r=1 and r=10) and a short justification for choosing these for emphasis. Local G*: You computed and mapped Z‑scores; consider summarizing notable clusters in the main text (currently in Supplement).*

**Response:** We selected *r* = 1 and *r* = 10 for Table 3 to represent the lower and upper bounds of our sensitivity analysis, respectively. These two values effectively capture the overall trend of the results (Supplementary Table S4). We revised the footnote of Table 3 (lines 443-446) to justify our choice.

As suggested, we have revised the paragraph describing the results of the local spatial statistic (Lines 449–452) to highlight notable clusters. This revision provides a clearer summary of the spatial risks within the main text, reducing the need for constant reference to the Supplementary information.

9. *Random CV (LOOCV, 5‑fold) can be spatially optimistic. Please add a spatially blocked CV sensitivity analysis (e.g., k‑fold by spatial clusters or spatial buffering) to re‑estimate AUC/TSS, at least in Supplement. Covariates: You considered 20 variables (+quadratics) from JAXA LULC (10 m), MLIT climate/topography (1 km), and mammals (5 km presence/absence). You then downscaled mammal layers uniformly across 1 km cells, which introduces pseudo‑precision; the manuscript should state this limitation clearly (you do briefly in Discussion—bring a sentence into Methods). Interactions: None were considered; add one sentence justifying this (to limit complexity and preserve power at N=171 sites).*

**Response:** We appreciate the reviewer’s insightful suggestion regarding the potential for spatial optimism in our cross-validation. While we agree that spatially blocked CV is a robust approach, implementing it effectively with our data was impossible with convergence. Therefore, rather than presenting unstable results, we have explicitly acknowledged this as a limitation in the Discussion (lines 625-628). We revised the text to emphasize that future studies with expanded datasets should employ spatially blocked CV and integrate additional occurrence data to establish more reliable predictions.
As for this possible pseudo‑precision of mammal distribution, we have added a sentence to the Methods section (lines 304–306) to mention it as follows: “Although the 5-km grid is the highest resolution available for mammal distribution data, uniform downscaling to 1 km might overlook fine-scale variations.”
We also revised the sentence on lines 319-321 to justify no interaction terms according to the suggestion.

10. *consider adding bootstrap CIs for these indices as a robustness check.*

**Response:** We appreciate the reviewer’s comment to provides a more robust assessment in our analysis. As suggested, 95% confidence intervals were calculated for both Schoener’s *D* and Warren’s *I* using a bootstrap approach with 1,000 replicates. The method was described on line 373-375 and the updated results were added in Table 4.

11. *Note that your mammal layers are coarse, and snow data are long‑term means; explicitly state these temporal and spatial mismatches.*

**Response:** Thank you for pointing out these data limitations. We have revised the sentences (on lines 565–571) incorporating the phrase “spatial and temporal mismatches” to acknowledge the constraints of our environmental predictors.

12. *Concise and faithful. Add one sentence on surveillance application (e.g., grids with high suitability for I. persulcatus overlapping with TBEV/pLDB/RFB are priorities), while reiterating low viral prevalence and model‑validation caveats.*

**Response:** We appreciate your thoughtful comment to improve our conclusion. We added a sentence according to the reviewer as follows (lines 644-647): “Although the predictions should be interpreted with caution due to the low viral prevalence and inherent limitations in the model-validation process, our results can help in prioritizing areas for future surveillance.”

13. *Provide a public dataset sufficient to reproduce the analysis, e.g.:1×1 km grid centroids (or jittered points within grid cells) for each site;per‑site tick counts (by species/sex/stage), pool metadata (pool size, Ct values, assay), and pathogen outcomes;processed environmental covariates or scripts to fetch and process them;R code for GLM/GAM, Moran’s I/G*, overlap metrics, and mapping. Deposit to Zenodo/Dryad/GitHub with DOIs and update the statement accordingly.*

**Response:** We fully agree with the importance of data transparency and reproducibility. However, due to concerns regarding potential reputational damage to the local communities and specific locations surveyed, we are unable to publicly release the exact or jittered GPS coordinates of the sampling sites. We have addressed your request by providing the following alternative datasets and resources:

First, the results of tick collection and pathogen screening at each site are provided in Supplementary Table S1. We have also added Supplementary Table S2, which provides the pool size and mean Ct values of positive samples, and cited it on line 395.

Furthermore, processed environmental covariates are provided in Supplementary Table S7, which was cited in Data Availability section. Finally, we have deposited the representative R code on GitHub (https://github.com/DRAM-IIZC/MebukiITO_Riskmap).

The Data Availability section was updated accordingly (lines 651-657).
