# Supplementary material for: Spatial distribution of ticks and tick-borne pathogens in central Hokkaido, Japan and associated ecological factors revealed by intensive short-term survey in 2024
Source: PLoS One. 2026 May 14;21(5):e0349386. doi: 10.1371/journal.pone.0349386 (PMC13175462; doi:10.1371/journal.pone.0349386)
Supplement: Attachment — Submitted filename: Response_to_Reviewers_auresp_3.docx [file pone.0349386.s016.docx]

**Response to editor comments**

We sincerely appreciate the editor’s efforts in providing point-by-point corrections to our manuscript. We have incorporated all the suggestions including the proper usage of articles. Furthermore, we have conducted a thorough grammatical review of the entire manuscript. Specifically, we removed the word "respectively" from several sentences to improve clarity and flow (Lines 167, 216, 399, 432, 544, and 584 in the clean version). We also revised some key sentences from passive voice to active voice (Lines 452-454, 550-552, and 636-638 in the clean version). All changes to the manuscript are marked by “Track Changes” option in 'Revised Manuscript with Track Changes'.

As for the comment “Line 15 (Short title): "tic k-borne" → "tick-borne" (spurious space)”, we have verified the Short Title and confirmed that "tick-borne" is correctly formatted without a space in our manuscript. We have re-saved the file to ensure this error does not appear in the final version.

**Correction to the calculation of Warrant’s *I*:**

We would like to inform the editor that we have corrected the formula used to calculate Warren’s *I*. In our original submission, we used the formula provided in the original article by Warren et al. (2008) [48]:

$I(p_{X}, p_{Y}) =1-\frac{1}{2}\sqrt{\sum_{i} {(\sqrt{p_{X,i}}-\sqrt{p_{Y,i}})}^{2}}$.

However, we identified that this specific formula contained an erratum, which was subsequently corrected by the authors in 2011 (<https://academic.oup.com/evolut/article/65/4/1215/6854212>). The correct formula is as follows:

$I(p_{X}, p_{Y}) =1-\frac{1}{2}\sum_{i} {(\sqrt{p_{X,i}}-\sqrt{p_{Y,i}})}^{2}$.

We have accordingly updated the Methods (line 369) and the results in Table 4. Crucially, since the correct formula is a monotonic transformation of the original one, this revision does not alter the relative rankings of the *I* values and, therefore, does not affect our claim in the manuscript.
